# Expression of *CsSCL1* and Rooting Response in Chestnut Leaves Are Dependent on the Auxin Polar Transport and the Ontogenetic Origin of the Tissues

**DOI:** 10.3390/plants12142657

**Published:** 2023-07-16

**Authors:** Elena Varas, Silvia Valladares, Jesús Vielba, Nieves Vidal, Conchi Sánchez

**Affiliations:** 1 Misión Biológica de Galicia Sede Santiago de Compostela, Consejo Superior de Investigaciones Científicas, Apdo 122, 15780 Santiago de Compostela, Spain; elenav@promiva.es (E.V.); svalladares@agromillora.com (S.V.); jmvielba@mbg.csic.es (J.V.); nieves@mbg.csic.es (N.V.); 2 Fundación Promiva, Ctra M-501, Km 5.4, Villaviciosa de Odón, 28670 Madrid, Spain; 3 Agromillora Iberia, C/El Rebato, s/n, 08379 Barcelona, Spain

**Keywords:** *Castanea sativa*, indole-3-butyric acid, in vitro leaves, maturation stage, N-1-naphthyl-phthalamic acid, *Scarecrow-like 1*, root regeneration

## Abstract

The mechanisms underlying the de novo regeneration of adventitious roots are still poorly understood, particularly in trees. We developed a system for studying adventitious rooting (AR) at physiological and molecular levels using leaves excised from chestnut microshoots of the same genotype but with two distinct ontogenetic origins that differ in rooting competence. Leaves were treated with auxin and N-1-naphthyl-phthalamic acid (NPA), an inhibitor of auxin polar transport (PAT). The physiological effects were investigated by recording rooting rates and the number and quality of the roots. Molecular responses were examined by localizing and monitoring the changes in the expression of *CsSCL1*, an auxin-inducible gene in juvenile and mature shoots during AR. The rooting response of leaves was ontogenetic-stage dependent and similar to that of the donor microshoots. Initiation of root primordia and root development were inhibited by application of NPA, although its effect depended on the timing of application. *CsSCL1* was upregulated by auxin only in rooting-competent leaves during the novo root organogenesis, and the expression was reduced by NPA. The inhibitory effect on gene expression was detected during the reprograming of rooting competent cells towards root initials in response to auxin, indicating that PAT-mediated upregulation of *CsSCL1* is required in the initial steps of AR in chestnut leaves. The localized expression of *CsSCL1* in the quiescent center (QC) also suggests a role for this gene in the maintenance of meristematic competence and root radial patterning.

## 1. Introduction

Adventitious rooting (AR) is an organogenic process that involves cell fate reprogramming to initiate a root meristem. In woody species, clonal propagation of elite trees is often hampered by a decline in rooting ability as the tree ages [1,2]. Although maturation is a major factor limiting the cloning of recalcitrant adult trees, it may be possible to regenerate adventitious roots from mature trees by using ontogenetically young tissues from the base of the tree [3,4,5].

For the development of adventitious roots and successful vegetative propagation, exogenous auxins are frequently used to initiate cell division and root primordia in plants, including woody species [6,7,8]. Indole-3-butyric acid (IBA), which is often used to induce adventitious roots in forest tree species, changes the expression of many genes at the whole plant level, although the gene response to auxin is highly dependent on the type of tissue [9] and it is specific to the cell receiving the auxin signal [10]. Canonical auxin signaling involves the perception of the hormone by the receptor complex formed by TIR1 and FB2, which leads to the degradation of Aux/IAA inhibitor proteins and releases Auxin Response Factors (ARFs), that will drive auxin-related gene expression [11,12]. However, increasing evidence suggests the existence of extranuclear perception mechanisms regulating auxin responses [13]. In the case of AR, the accumulation of asymmetrically distributed auxin in specific cells is mandatory to tag them as root founder cells, reprogramming their genetic program and entering a root developmental pathway [14]. This asymmetric distribution is the result of the activity of the Polar Auxin Transport (PAT) machinery, which includes several families of transport proteins [15]. Therefore, modulation of PAT through the application of exogenous auxin or by means of specific PAT inhibitors enables the induction of AR or helps in the understanding of the molecular mechanisms related to the induction of the process, respectively. N-1-naphthyl-phthalamic acid (NPA) is a PAT inhibitor widely used in research to characterize auxin-related responses and in horticulture and agriculture as an anti-auxin compound [16].

Different model systems have been used to study phase change and the maturation- or age-dependent decline of rooting competence, including leaf petioles from ivy plants [17,18], cuttings from hypocotyls and epicotyls of *Pinus taeda*, *P. radiata* and Arabidopsis seedlings [2,19,20,21], juvenile and mature cuttings from eucalyptus [22] and black walnut [23] or cuttings from pea plants of different ages [24].

On the basis of the juvenile-mature gradient displayed in plants, an experimental system was developed with microshoots derived from basal sprouts and crown branches of the same chestnut tree [1]. Both juvenile and mature shoot lines exhibited different in vitro morphogenetic capacity and were used to study phase change and the maturation-related decline of AR [5,25,26,27,28]. This system allowed for the identification of the *Castanea sativa Scarecrow-like 1* gene (*CsSCL1*), encoding a transcription factor from the GRAS family, which was upregulated by auxin within the first 24 h of treatment [20]. GRAS proteins are involved in cell division, auxin signaling, root radial patterning and root meristem specification, among other processes [29,30,31]. The localized expression of the gene in rooting competent cells of juvenile shoots indicates that *CsSCL1* plays an important role in the initiation of adventitious roots [27]. Auxin-induced expression of *SCL1* was also detected in root progenitor cells only in juvenile cuttings during the AR of black walnut [23]. Although the microshoot system is suitable for identifying rooting markers, the complexity of the shoots, in which several processes occur simultaneously in response to the auxin treatment, can sometimes create difficulties in the analysis of the results or mask the pursued responses. Auxin distribution at the base of wounded shoots, where exogenous auxin is applied to induce roots, may be altered by PAT from the shoot apex. For example, IAA levels during rooting were not positively correlated with the AR ability in juvenile and mature shoots of chestnut [25] and oak [32]. Therefore, the development of simplified working systems might be useful for the detailed analysis of the relation between PAT and AR. Ideal systems should comprise only the essential tissues implied in the process, thus reducing the number of incidental physiological responses that could add background noise to the studies and difficult interpretation. Our main goal was to devise and validate a working system achieving those premises.

Here we describe for the first time in chestnut a simple and useful leaf-based system to study adventitious rooting, taking into consideration auxin signaling and transport, as well as the loss of rooting competence on maturation. Auxin-induced expression of *CsSCL1* was only detected in rooting competent leaves. NPA inhibition of PAT revealed a direct correlation between NPA-induced reduction of AR capacity and a down-regulation of the auxin-induced expression of *CsSCL1*, also associated with rooting competent cells.

## 2. Results

### 2.1. Leaves Exhibited a Similar Rooting Response to the Donor Shoots

With the aim of developing a practical and simple experimental system in chestnut for studying AR, we evaluated the rhizogenic responses of microshoots, leaves and leaf segments from the P2BS (Basal Sprouts, juvenile-like) line. Treatment with 25 µM IBA for 5 days under dark conditions was chosen from preliminary experiments and used to induce adventitious roots in leaves and leaf segments

**Figure 1:**
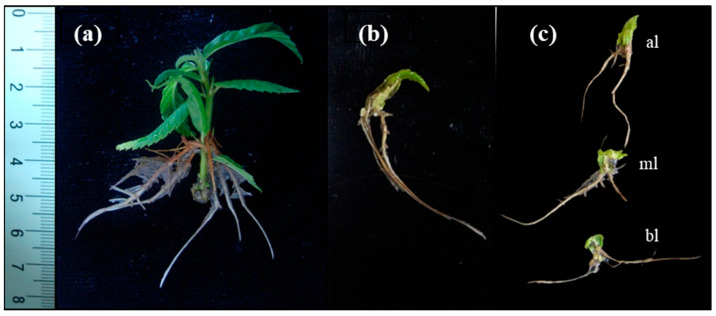
Root development in chestnut explants derived from basal sprouts (P2BS) 30 days after beginning of treatments. (**a**) Microshoots were dipped in 4.9 mM IBA solution for 1 min and then placed on IBA-free medium. (**b**) Leaves excised from microshoots were placed for 5 days on medium containing 25 µM IBA and then transferred to IBA-free medium. (**c**) The three different leaf segments, apical (al), middle (ml) and basal (bl), were treated as described for leaves.

Whole leaves (including petiole) excised from juvenile microshoots exhibited similar rooting rates as the donor microshoots, whereas the use of leaf segments as the explant source led to a significant decrease in rooting rates (Table 1). No differences were observed in the rooting response between the three different types of leaf segments used in the experiment. Mean rooting time ranged from 9 to 11 days in all three types of explants. The mean number of roots was significantly higher in microshoots (8.4) than in whole leaves (5.8) and leaf segments (2.9). This parameter was significantly lower in leaf explants than in the other tested explants. By contrast, root length was significantly longer in leaves than in microshoots and leaf segments. Differences were also found in the root patterning of microshoots and leaves, which was determined by the number of roots that developed on either the basal part of the explant or outside of that area. In microshoots, 52% of the roots formed along the stem and exhibited acrobasal development (Figure 1a). On the other hand, in leaf explants most of the roots emerged from the cut end of the petioles/segments (91 to 97%) in contact with the medium and from the mid-vein near the wound (Table 1, Figure 1b,c). In leaves, roots emerged without callus formation; however, callus grew around the wounded area. As the rooting response to IBA was lower in leaf segments than in leaves, the former were discarded in further experiments.

To test whether this leaf system could also be used to study the loss of rooting ability associated with maturation, we compared the rooting ability of microshoots and leaves from the P2CR (Crown, mature) line. In both CR microshoots and leaves, the rooting rate was less than 10%, whereas in BS-derived explants the average rooting rate was 95% (see Supplementary Figure S3). Furthermore, materials of the same origin also exhibited similar rooting kinetics. Root emergence was observed in BS leaves and shoots as early as 7 days after treatment, whereas in mature explants the first root emerged between 25 (leaves) and 29 days (shoots) (see Supplementary Figure S3). These results confirm that the rhizogenic ability of leaves is also dependent on the ontogenetic stage of the mother shoot.

### 2.2. The Inhibitory Effect of NPA on Rooting Ability of Chestnut Depends on the Timing of Application

In the proposed system, the application of exogenous IBA is critical for triggering the rooting process. We therefore studied the effect of NPA on the rooting response of both microshoots and leaves from BS line treated with IBA.

**Figure 2:**
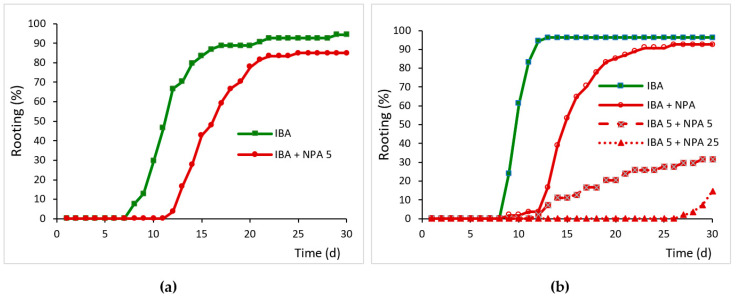
Effect of NPA on the rooting response of juvenile chestnut microshoots and leaves. (**a**) Microshoots were dipped in 4.9 mM IBA for 1 min and then placed on 1/3 GD medium without NPA (IBA) or with 50 µM NPA for 5 days (IBA + NPA 5). (**b**) Leaves were treated with 25 µM IBA and transferred to IBA-free medium after 5 days (IBA). In addition, 50 µM NPA was added simultaneously to IBA treatment (IBA + NPA), during the 5 days following IBA treatment (IBA 5 + NPA 5) or during the 25 days following IBA treatment (IBA 5 + NPA 25).

No significant differences were observed in the rooting percentage of microshoots treated or not treated with NPA (87% vs. 94%); however, the treatment delayed the onset of root emergence as well as the mean rooting time by 4 days (Figure 2a). In leaves, the simultaneous application of IBA and NPA for 5 days significantly reduced the rooting percentage and increased the mean rooting time relative to those induced by the IBA treatment (Figure 2b). Mean rooting time was increased by 4.4 days in shoots dipped in IBA solution and then transferred to NPA-containing medium, whereas in leaves treated simultaneously with IBA and NPA it increased to 8.5 days. These data showed that NPA supplied at the same time as auxin inhibited the IBA-induced root initiation in juvenile leaves.

The effect of NPA on root development was tested by transferring leaves treated with IBA for 5 days to an NPA-containing medium. Rooting percentage was not affected by NPA when it was applied after the IBA treatment (Figure 2b). However, mean rooting time also increased by 6.3 days, as observed in the microshoots treated with IBA and NPA. By increasing the length of post IBA treatment with NPA to 25 days, there was a clear negative effect on all the parameters evaluated. Rooting rates were significantly lower in leaves after the 25 day-NPA treatment, when compared to the IBA-treated leaves. Roots appeared after 27 days of root induction, and the roots were very short, as the data were evaluated after 30 days. Application of NPA for 25 days strongly inhibited root emergence, indicating that NPA affects root primordia development.

**Figure 3:**
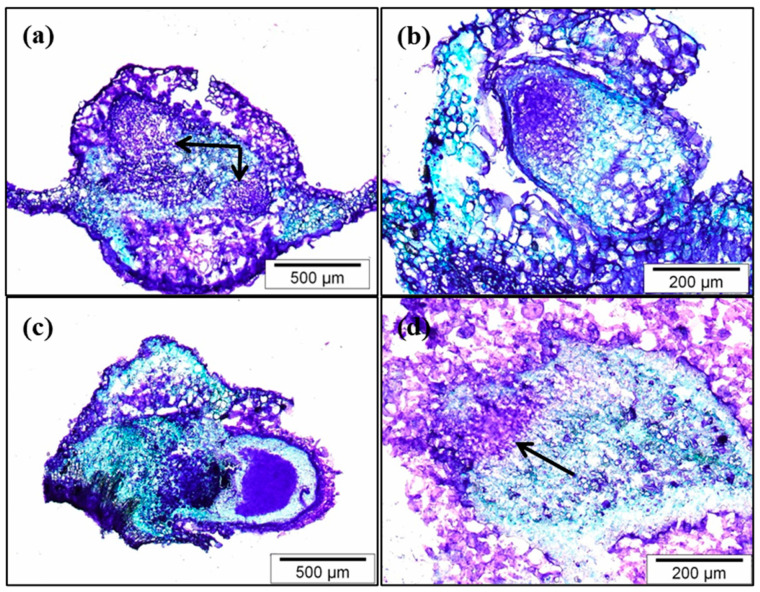
Effect of NPA on the development of adventitious roots in leaves 10 days after the start of treatments. (**a**,**b**) Leaves were treated with 25 µM IBA for 5 days and then transferred to IBA-free medium. (**c**) Leaves were simultaneously treated with 25 µM IBA and 50 µM NPA for 5 days. (**d**) Leaves were treated with 25 µM IBA for 5 days and then transferred to IBA-free medium supplemented with 50 µM NPA for 5 days. Sections of 10 µm were stained with toluidine blue. Root primordia in (**a**) and the incipient meristem in (**d**) are indicated by arrows.

Histological observations showed the inhibition of adventitious root formation in leaves treated with NPA. By 10 days after the start of the experiment, leaves treated with IBA showed incipient roots or dome-shaped root primordia (Figure 3a,b).

In leaves treated simultaneously with IBA and NPA, root primordia were only occasionally found after 10 days, as the rooting percentage was very low and the rooting time increased by more than 5 days. In this treatment, roots had regenerated from the cut surface of the basal petiole (Figure 3c), whereas in the IBA treatment they formed about 1–2 mm above the cut surface. However, when root primordia were initiated, they appeared to be at a similar developmental stage as those in IBA-treated leaves (Figure 3c). In leaves treated simultaneously with IBA and NPA (IBA + NPA), the few primordia that developed during the induction period appeared at the base of the petiole, following a normal development, although the tip was rounded (Figure 3c). Development of root primordia was negatively affected and delayed when IBA-treated leaves were transferred to NPA-containing medium, and only a group of meristematic cells was observed at 10 days (Figure 3d), in concordance with a 6.3 day delay in the mean rooting time. These meristemoids seemed to be less compacted and more disorganized than those that appeared in IBA-treated leaves. Application of NPA to IBA-treated leaves appeared to promote callus formation around the incipient root primordia and arrest its development, which then resumed after the NPA was removed from the medium, as indicated by the similar rooting rates at the end of rooting period and in the IBA treatment (Figure 2b).

The above data indicated that the NPA negatively affected root initiation and root emergence and that the effects are also dependent on the timing and period of application.

### 2.3. CsSCL1 Is Highly Expressed in Roots

In a previous study, we evaluated the levels of *CsSCL1* expression in different organs of chestnut microshoots [20]. In the present work, we carried out a more detailed analysis with the aim of determining the basal expression levels of the gene in leaves excised from microshoots at the end of the multiplication period. Furthermore, we checked the relative expression levels of the gene in the meristematic, elongation and maturation zones of the root. Internodes and axillary buds were also included in this analysis (see Supplementary Figure S2).

**Figure 4:**
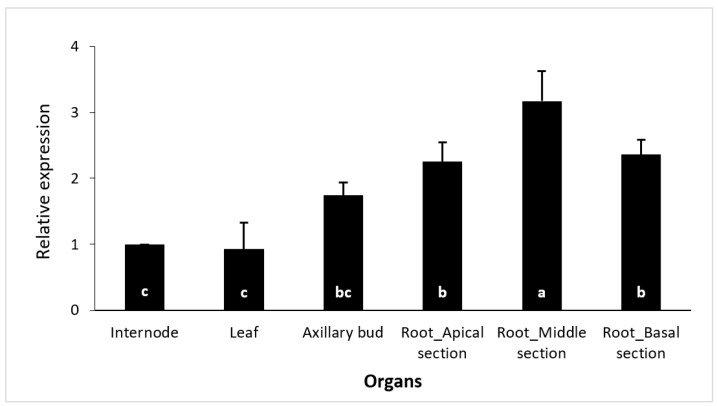
Expression analysis of *CsSCL1* by qPCR in different organs: internode, leaf, axillary bud and the apical, middle and basal sections of the adventitious root. Results are expressed as mean values ± SE of three biological replicates, and the relative expression was normalized to shoot internode levels. The same letter at the bottom of the bars indicates no significant difference at *p* ≤ 0.05.

In general, *CsSCL1* transcripts accumulated to relatively higher levels in roots than in the other organs (Figure 4). The relative expression level was highest in the middle area of the root, and it was significantly different from those in the apical and basal parts of the root. The expression levels were lowest in internodes and leaves. These results indicated that leaves may be suitable for studying the regulation and patterns of expression of auxin inducible genes such as *CsSCL1* during AR, as the basal expression levels are relatively low.

### 2.4. CsSCL1 Is Induced by Auxin Only in Rooting-Competent Leaves

To explore the suitability of the leaf system for studying changes in gene expression associated with the transition from an incompetent state to competence in the root organogenesis induced by auxin, we evaluated *CsSCL1* expression during the earliest steps of adventitious root induction (6, 12 and 24 h).

**Figure 5:**
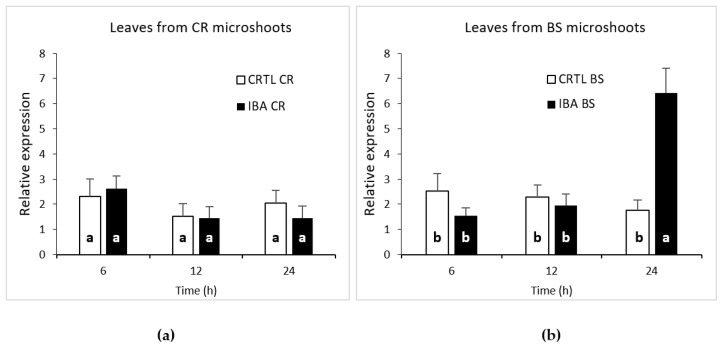
Expression of *CsSCL1* during adventitious root induction of leaves excised from microshoots derived from crown branches (**a**) and from basal sprouts (**b**). Leaves were treated with 25 µM IBA (IBA) or were not treated (CTRL) and harvested at 6, 12 and 24 h. The different letters (a, b) at the bottom of the bars indicates significant difference at *p* ≤ 0.05. The increase in gene expression at 24 h after IBA treatment in BS leaves was significant at *p* ≤ 0.05. The interaction time × treatment was significant at *p* ≤ 0.001.

The results showed that *CsSCL1* levels were not significantly affected (*p* ≤ 0.05) either by wounding or by exogenous auxin in rooting-incompetent leaves within the first 24 h (Figure 5a), as steady state levels of transcripts remained constant in control and IBA-treated samples. In contrast, a significant increase (*p* ≤ 0.05) in the relative levels of *CsSCL1* expression was detected in IBA-treated juvenile leaves 24 h after treatment, whereas no differences were observed after 6 or 12 h between treated and control leaves (Figure 5b). These results indicated that *CsSCL1* is strongly induced by exogenous auxin only in juvenile leaves that are rooting-competent in response to IBA.

### 2.5. NPA Reduces Peak Expression of CsSCL1 during Induction of Adventitious Roots

**Figure 6:**
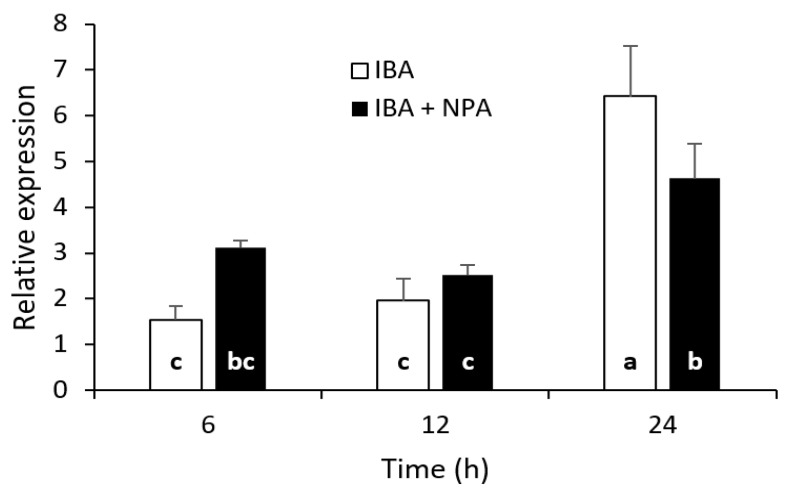
Effect of NPA on *CsSCL1* expression during adventitious root induction in leaves excised from P2BS microshoots. Leaves were treated with 25 µM IBA (IBA) or simultaneously with IBA and 50 µM of NPA (IBA + NPA) and harvested 6, 12 and 24 h after treatment. The same letter at the bottom of the bars indicates no significant difference at *p* ≤ 0.05. The interaction time × treatment was significant at *p* ≤ 0.05.

We observed a strong effect of NPA on the dynamics and rooting rates of AR (Figure 2b). Therefore, to investigate whether the concomitant application of NPA and IBA affects *CsSCL1* expression, we analyzed the transcript levels of the gene in juvenile IBA-treated leaves (supplemented or not with NPA) during the first 24 h of root induction (Figure 6).

Simultaneous treatment of rooting-competent leaves with IBA and NPA led to a significant decrease in *CsSCL1* transcript levels in IBA-treated leaves 24 h after treatment. The data revealed a direct correlation between the negative effect of NPA on the expression of *CsSCL1* in IBA-treated leaves and inhibition of AR (Figure 2b). Interestingly, NPA had a slight inductive effect on the expression of the gene as soon as 6h after the beginning of the treatment.

### 2.6. Distribution of CsSCL1 Transcripts Is Affected by Maturation and by NPA during the Induction of Adventitious Roots

To determine whether the different auxin-induced levels of *CsSCL1* transcripts detected in BS and CR leaves and the reduction of *CsSCL1* expression by NPA are associated with specific cell types involved in AR, we conducted in situ expression experiments.

**Figure 7:**
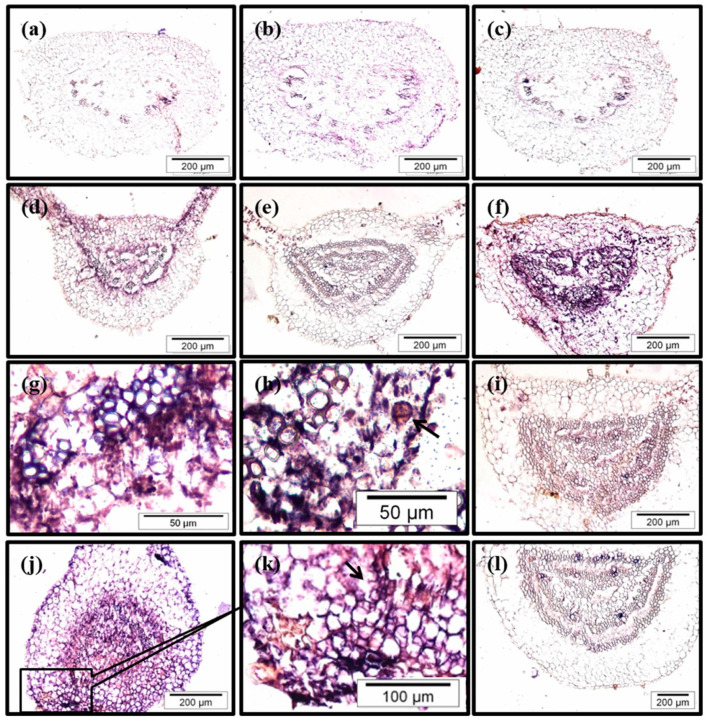
In situ localization of *CsSCL1* mRNA in sections of leaves excised from crown (**a**–**c**) and basal (**d**–**l**) microshoots. Leaves were treated with 0 µM IBA (**b**,**e**), 25 µM IBA (**a**,**c**,**d**,**f**–**h**,**j**,**k**) or with 25 µM IBA and 50 µM NPA simultaneously (**i**,**l**). Leaves were harvested 12 h (**d**), 24 h (**a**–**c**,**e**–**i**,**l**) or 72 h (**j**,**k**) after the start of treatments. Sections were hybridized with the antisense probe (**b**–**k**) or the sense probe (**a**,**l**). *CsSCL1* expression is localized to cells surrounding the vascular tissues in IBA-treated juvenile leaves (**d**,**f**,**g**). Putative asymmetric cell division (**h**) and organized cell divisions (**k**) are indicated by arrows.

We found important differences in the patterns of *CsSCL1* expression between CR and BS leaves 24 h after treatment with IBA (Figure 7c compared with Figure 7f). In CR leaves, harvested after 24 h of culture in IBA medium or in IBA-free medium, *CsSCL1* transcripts produced only a diffuse signal in all tissues (Figure 7c), as observed in samples cultured in IBA-free medium (Figure 7b,e). By contrast, in BS leaves the strongest signal was detected after the longest period of IBA treatment (72 h; Figure 7d,f,j). This signal was localized in the cambium, phloem cells and interfascicular parenchyma cells surrounding the vascular tissue (Figure 7d,g). No signal was detected in either the untreated-IBA leaves (Figure 7b,e) or in samples hybridized with the sense probe (Figure 7a,l). Low levels of transcripts were detected in rooting competent cells of BS leaves treated simultaneously with IBA and NPA, when compared to leaves treated only with IBA (Figure 7i compared with Figure 7f), in concordance with the previous findings of the qPCR analysis (Figure 6).

In IBA-treated juvenile leaves, asymmetric cell divisions were occasionally observed after 24 h (Figure 7h) and probably gave rise to root initial cells. After 72 h of treatment with IBA, the number of organized cell divisions increased in the phloem area, and they presumably generated the new root primordia (Figure 7j,k). Abundant mRNA levels were detected in the cambium cells and phloem cells of the petioles during cell reorganization and prior to cell divisions (Figure 7d) and also in the phloem area and parenchyma cells surrounding the vascular tissue. These cells undergo active cell division at 72 h and probably give rise to the root meristem (Figure 7j,k).

The above results showed that *CsSCL1* expression is regulated in a rooting competent-dependent manner during the early stages of AR and is associated with rooting-competent cells.

### 2.7. CsSCL1 Is Expressed in the Root Primordia and the Quiescent Center of the Root

**Figure 8:**
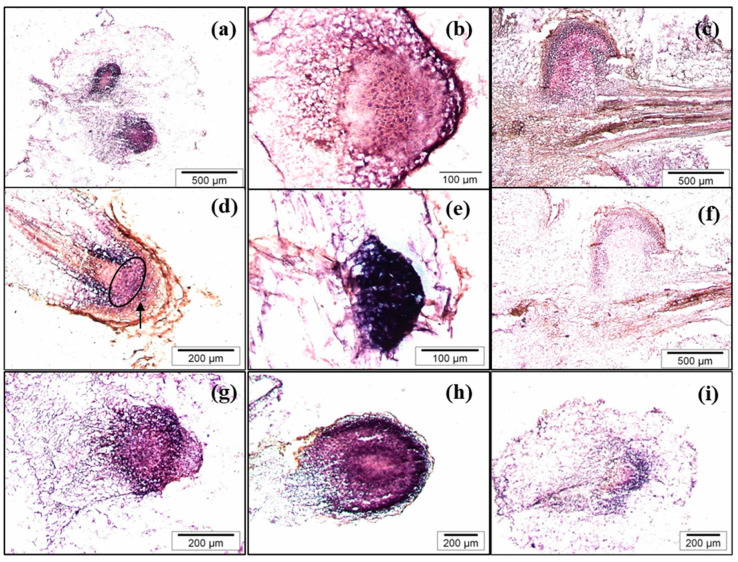
In situ localization of *CsSCL1* mRNA in cross (**a**,**b**,**f**–**i**) and longitudinal sections (**c**–**f**) of roots developed from BS leaves. Leaves were treated with IBA (**a**–**g**), treated simultaneously with IBA and NPA (**h**) or treated with IBA and then transferred to NPA-containing medium (**i**). Sections were hybridized with the antisense (**a**–**e**,**g**–**i**) or the sense probe (**f**). The arrow in (**d**) indicates the columella progenitor cells.

In situ experiments showed that the gene is strongly expressed in the root primordia (Figure 8a–c) and in the lateral root primordia developed along the adventitious root (Figure 8e). As shown in the longitudinal section of IBA-treated petioles harvested after 10 days of culture, roots clearly originated from the vascular tissue (Figure 8c). At a more advanced developmental stage of the root, the gene is also expressed in the quiescent center (QC) of the root tip, as well as in the cortex/endodermis and root cap initial cells and their derivatives (Figure 8d). Expression also occurs in the columella progenitor cells.

In IBA-treated leaves, by day 10, a strong signal was detected in all cells of the dome-shape root primordia with the incipient columella (Figure 8g). A similar expression pattern was also detected in the round-shaped primordia, with no evidence of initial columella, which were only occasionally observed after 10 days in leaves treated simultaneously with IBA and NPA (Figure 8h). In IBA-treated leaves that were then transferred to NPA medium for 5 days, a lower and more diffuse hybridization signal was only observed in the peripheral cell of the meristemoid after 10 days, relative to the strong signal detected in the root meristem developed from IBA-treated leaves (Figure 8i compared with Figure 8g). Therefore, NPA disrupts the root development as well as the pattern and levels of *CsSCL1* expression. These data suggest that *CsSCL1* participates in controlling the radial patterning of the root as it is expressed in the QC.

### 2.8. Usefulness of the Leaf Rooting System in Different Woody Species

**Figure 9:**
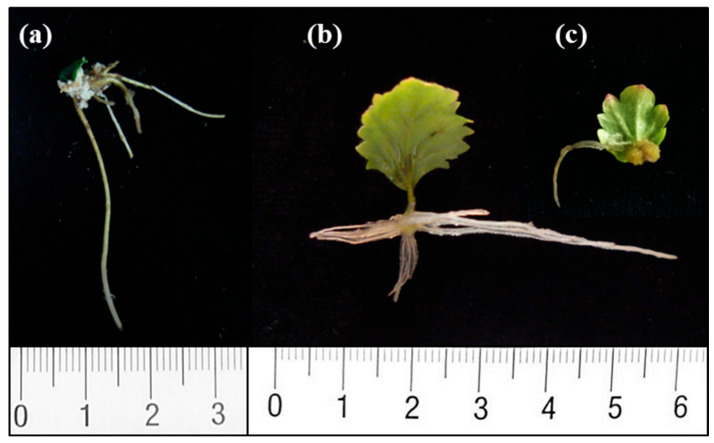
Adventitious rooting in leaves excised from in vitro proliferating shoots of cork oak (**a**), and birch (**b**,**c**). (**a**,**b**) Leaves were treated with 25 µM IBA for 5 days and then transferred to IBA-free medium. (**c**) Adventitious root formation in birch leaf not treated with IBA.

To investigate the usefulness of this system in other woody species (easy- and difficult-to-root species), detached leaves from shoot cultures established from mature birch and cork oak trees were used. After treatment with IBA, roots were regenerated in both species by using the same auxin treatment described in chestnut (Figure 9a,b). Differences in root number and length were observed between leaves of both species. The number of roots regenerated per leaf was greater in birch leaves (about 10–13 roots) than in cork oak leaves (2–5). By contrast, the average root length was slightly longer in cork oak than in birch. In the case of birch, roots were also regenerated from leaves without IBA treatment (Figure 9c), thus providing an alternative model for studying the effect of endogenous auxin during AR. The leaf rooting system can therefore be used in the tested species to study AR.

## 3. Discussion

In the present study, we developed a simple method using leaves excised from shoots to study the histological and molecular mechanisms involved in regenerating adventitious roots. We demonstrated that the rooting rates in the leaves and the mother shoots from which they were excised were similar and correlated with the ontogenetic origin, consistent with the data previously reported in microshoots [1,25]. The decline in the morphogenetic potential of woody species with increasing physiological age and ontogenetic state during the ongoing process of phase change has been observed in various studies [3,33,34,35]. The strong influence of ontogenetic stage on rooting ability has also been demonstrated in pea cuttings [24], and in chestnut it has been linked to changes in gene expression [28,36,37]. The leaf system described here increased the potential number of samples by at least three times and also involved the use of less complex tissues, since roots arose from a small area at the base of the leaf petiole. In microshoots, roots regenerated from the basal part of the shoot and also along the stem. Thus, leaf system also prevents the potential side-effects caused by PAT in the shoot whereby endogenous auxin moves basipetally from the shoot apex and excludes other auxin-modulated developmental processes that may take place simultaneously in the shoot after IBA treatment. Overall, these advantages make the system highly suitable for studying the molecular mechanisms underlying adventitious root formation. Leaf explants have also been used to study AR in *Medicago truncatula* [38]. Different leaf protocols in Arabidopsis have been developed for studying AR, in which roots can be regenerated without auxin treatment [39]. However, in our system, exogenous auxin is required to regenerate adventitious roots, as in most difficult-to-root woody species. In a leaf Arabidopsis system, it was shown that young leaves present a higher regeneration ability than mature ones [40], which is in agreement with our findings in the chestnut leaf system.

Differences in regeneration ability between plants with contrasting ontogenetic state are believed to rely on distinct epigenetic status and hormone signaling, with the intertwined combination of both governing plant developmental plasticity and other processes [41]. In a recent report, significant differences in hormone- and epigenetic-related signaling were found between auxin-treated chestnut microshoots with different ontogenetic state [37]. Moreover, an age-related signaling pathway involving miRNAs, and the *Squamosa Promoter Binding Protein-like* (SPLs) genes govern the transition from the juvenile to the mature stage. High expression of *miR156* during the juvenile period targets *SPL* genes for degradation, with eventual lower levels of the miRNAs allowing the expression of *SPLs* and permitting the transition to the mature stage (reviewed in [42]). Whether this age-related pathway or other mechanisms, such as histone modifications or hormone sensitivity, are linked to the differences found in the present work remains to be elucidated.

In a previous study with chestnut microshoots, we demonstrated that *CsSCL1* is induced by auxin in juvenile and mature microshoots and is specifically expressed in cells involved in root initiation only in rooting competent shoots [27]. In the present study, we demonstrated that *CsSCL1* expression is only induced in rooting-competent leaves, probably due to a reduced auxin signaling in mature detached leaves. We also described the specific distribution of mRNA transcripts associated with the activation of cells involved in initiating root meristems. By reducing the complexity of explants induced to root and excluding other auxin-regulated processes that occur in microshoots, auxin-induced *CsSCL1* expression was only detected in the juvenile material, whereas the gene was also upregulated by auxin in mature microshoots (compare Figure 5 with Figure 3B,C in [27]). Moreover, *CsSCL1* expression was induced when cell reorganization occurred and, occasionally, when the first asymmetric cell divisions were observed. The direct relationship between rooting ability and gene expression simplifies the molecular analysis and data interpretation. Expression of *SCL1* homologs has also been related to the AR process in pine, black walnut and eucalyptus, suggesting its role as a marker of rooting ability in forest species [20,23,43]. The localized expression of *CsSCL1* in the QC and stem cells also indicates the involvement of the gene in maintaining the radial patterning of the root. This is also supported by the lack of an incipient columella in the round-shaped primordia developed after NPA treatment. The regulatory role of the GRAS family genes *SCR* and *SHR* in root radial patterning and maintenance of QC identity has been well documented [44,45,46].

The present study also provides evidence that the effect of NPA on the rooting response is highly dependent on the timing (when and for how long) of the treatment. The onset of AR was always delayed by NPA, regardless of the type of treatment. A similar response has been observed in other species such as pine, oak and grapevine [32,47,48]. In addition, auxin-induced activation of target-competent cells was not greatly impaired by the temporal (5 day) post-application of NPA, as once NPA was removed from the medium the cells were able to resume division and proceed to the rooting pathway. However, root emergence was prevented by the subsequent and continuous treatment with NPA (25 days) after the IBA treatment. This demonstrates that NPA not only inhibited the root primordia initiation but also root emergence and development, indicating a role for auxin transport in those processes. Moreover, the differentiation of rooting-competent cells to root initial cells was strongly inhibited by NPA when applied at the same time as IBA. It appears that NPA impaired the creation of an optimal auxin gradient in the target cells required for initiation of root meristem. In Arabidopsis, a maximum auxin gradient in the root stem cell niche is crucial for proper root development and patterning [49,50]. Finally, we demonstrated for the first time the NPA-mediated effect on gene expression during the adventitious rooting of chestnut leaves. The inhibitory effect of NPA on root initiation was correlated with the reduced levels of *CsSCL1* expression observed in leaves 24 h after simultaneous treatment with IBA and NPA, and the spatial expression pattern also varied in NPA-treated leaves. These data confirm our previous findings regarding the involvement of *CsSCL1* in AR [27], as well as the sensitivity to NPA that alters PAT and probably disrupts or impedes the creation of the auxin gradient required for the initiation of a root meristem. In Arabidopsis, gene expression related to de novo root regeneration from leaves was blocked or impaired as a consequence of NPA treatment [51].

The function of auxin signaling and transport in adventitious root formation have been reported in different species [2,52,53,54]. Inhibitors of plant auxin transport, such as NPA, were shown to delay and inhibit adventitious root formation [32,48,49,55,56,57]. A direct correlation between inhibition of AR by NPA and suppression of the 24 h peak of IAA during induction of adventitious roots was also demonstrated in petunia [58]. In the model system described in the present work, NPA activity delayed and/or inhibited AR, depending on the mode of application. Therefore, blocking of PAT impedes the establishment of a new developmental program, although not irreversibly.

The ability of NPA to inhibit PAT has been known for a long time, and several possible explanations have been proposed [59]. However, only recently the molecular basis of this process has begun to be unraveled. Several protein families are known to have the capacity to transport auxins, including PIN, PILs and ABCBs [15], and they could therefore be potential targets of the NPA activity. Nonetheless, PAT-related auxin efflux from cells occurs by means of the activity of PIN proteins, a family of polarly localized transmembrane transporters. NPA was shown to specifically inhibit PIN1 mediated auxin transport [60], while other report demonstrated that, once inside the cell, NPA can bind to different members of the PIN family through direct interaction with their inner domains, blocking their activity [61]. Indeed, the activity of specific PIN proteins in the initial stages of AR has been shown in different species, like apple or olive tree [62,63]. In mature chestnut microshoots, increased activity of *CsPIN1* has been linked to an improved rooting response (personal communication R. Castro-Camba). Therefore, although other related action mechanisms cannot be ruled out, NPA might be blocking the activity of specific members of the PIN family in chestnut, impeding the establishment of auxin gradients. These gradients and the generated maxima and minima are essential in plants to deploy plastic developmental responses [16].

## 4. Materials and Methods

### 4.1. Plant Material

Stock shoot cultures of chestnut initiated from basal sprouts (BS) and crown branches (CR) of the same tree (P2 clone) were used in the study. The techniques for in vitro establishment and proliferation of shoots have already been described [1]. Both lines have been maintained in vitro in our laboratory for more than 20 years, and they retain different morphological characteristics as well as different rooting abilities associated with their maturation stage.

### 4.2. Adventitious Roots Induction

Rooting experiments were performed on (1) shoots of 2.5 to 3.5 cm in length, harvested from 4-week-old proliferating cultures growing in multiplication medium, (2) the three youngest and fully expanded leaves with petioles detached from the upper thirds of these microshoots and (3) leaf segments, to compare their rooting response versus whole leaves. Whole leaves were placed on the medium abaxial side down, with the cut ends of the petioles inoculated in the medium (see Supplementary Figure S1). Leaf segments were obtained from leaves that were cut transversely through the mid-vein into three segments: the basal part including the petiole, the middle segment and the upper part containing the leaf apex. The polarity of leaf segments was maintained, and they were placed upright on the medium, with the cut surface in contact with the medium.

Root induction on shoots was performed by dipping the basal end of each shoot in 4.9 mM of IBA for 1 min. The shoots were subsequently transferred to 1/3-strength macronutrients Gresshoff and Doy [64] medium (1/3 GD) [1]. Leaves and leaf segments were placed on 1/3 GD medium supplemented with 0 (control) or 25 µM IBA for 5 days in darkness before being transferred to IBA-free medium under a 16 h photoperiod.

**Figure 10:**
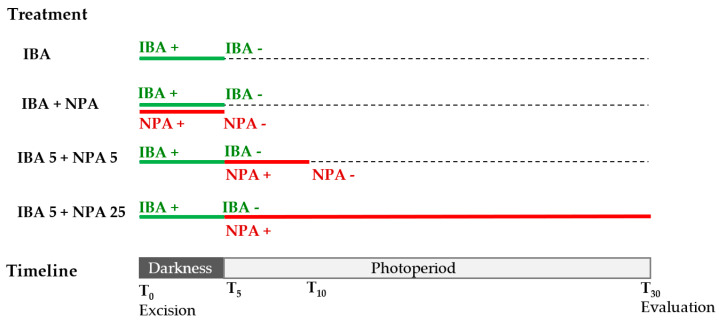
Indole-3-butyric acid (IBA) and N-1-naphthyl-phthalamic acid (NPA) treatments applied to chestnut leaves. Leaves excised from chestnut microshoots were treated with 25 µM IBA for 5 days in darkness and transferred to IBA-free medium under normal photoperiod. NPA (50 µM) was applied simultaneously to IBA treatment for 5 days or after IBA treatment for 5 or 25 days. Evaluation was performed at day 30. IBA+, application of IBA; IBA−, transference to IBA-free medium. NPA+, application of NPA; NPA−, transference to NPA-free medium.

The effect of NPA on rooting was studied in both microshoots and leaves of the BS line. Microshoots treated with IBA were placed in 1/3 GD medium supplemented with 50 µM NPA for 0 (IBA) or 5 days (IBA 5 + NPA5). The effect of NPA on root development was tested in auxin-treated leaves by applying 4 treatments designated as IBA, IBA + NPA, IBA5 + NPA5 and IBA5 + NPA25 (Figure 10). Leaves were placed on 1/3 GD medium supplemented with 25 µM IBA for 5 days in darkness, and they were subsequently transferred to IBA-free medium under normal photoperiod (IBA). Following the same procedure, leaves were treated with 50 µM NPA simultaneously to IBA treatment and transferred to auxin- and NPA-free medium (IBA + NPA) or treated with NPA after IBA treatment for 5 (IBA5 + NPA5) or 25 days (IBA5 + NPA25).

Rooting rate (%), number of roots per rooted explant, % of non-basal roots, the longest root length, the time elapsed before the appearance of the first root and the mean rooting time were recorded 30 days after the beginning of the experiment. Rooting time was evaluated as the mean day of root emergence of rooted explants. Leaf samples were also collected 6, 12, 24 and 72 h after each treatment for RNA extraction and/or in situ hybridization.

### 4.3. RNA Extraction and Quantification

Plant material was harvested, frozen in liquid nitrogen and stored at −70 °C until use for RNA extraction. Axillary buds, internodes and leaves excised from BS microshoots were harvested at the end of the proliferation cycle. Adventitious roots were harvested 30 days after rooting induction and divided into three segments: apical (0–1 cm), middle (1–3 cm) and basal (3–4 cm) portions (see Supplementary Figure S2). IBA-treated leaves, control leaves and NPA-treated leaves were harvested 6, 12 and 24 h after treatments. Total RNA was extracted using the FavorPrep Plant Total RNA purification Mini Kit (for woody Plant) according to the manufacturer’s instructions.

### 4.4. Quantitative Reverse Transcriptase-Polymerase Chain Reaction (qPCR)

For each sample, first strand cDNA synthesis was performed with 1 µg of total RNA in a final volume of 20 µL using the High Capacity cDNA Reverse Transcription kit (Applied Biosystems) according to the manufacturer’s instructions. The *CsSCL1* primers used in this study were previously designed for the analysis of chestnut microshoots [27]. Three reference genes, Actin (ACT), Tubulin (TUB) and Polyubiquitin (UBI), were used as internal controls to normalize all data. The specificity of the primers and amplifications was confirmed by PCR and sequencing of the amplicons. The concentration of primers was adapted to each experiment, and primer efficiency was tested using a standard curve for each gene. The genes, accession numbers, primer sequences and the amplicon length are outlined in the Supplementary Materials (see Supplementary Table S1).

The qPCR analyses were carried out in an optical 48-well reaction plate with a StepOne Real-Time PCR System (Applied Biosystems) and with SYBR Green Master Mix (Applied Biosystems) to monitor PCR amplification. Reaction mixtures contained 1 X Power SYBR^®^ Green and 8.3 ng of cDNA as template, in a total volume of 15 µL. The concentration of *CsSCL1* primers was adjusted to 725 nM and 600 nM for the analyses carried out with samples of the different organs and from root induction experiments, respectively. Reaction mixtures were incubated at 95 ºC for 10 min, followed by 40 cycles of 95 °C for 15 s and 60 °C for 1 min. Three independent biological samples were used, and expression levels in each sample were based on 3 technical replicates. For quantification of *CsSCL1* in different organs, the results were expressed relative to the expression in internodes. In rooting experiments, data were expressed relative to the sample with lowest expression level in each biological replicate. Relative *CsSCL1* expression was expressed as a fold-change, as determined by the comparative Ct method [65]. All calculations and normalizations were performed using DataAssist™ v3.0 software (Applied Biosystems, Foster City, USA).

### 4.5. In Situ Hybridization and Histological Analysis

To analyze the *CsSCL1* expression pattern during the root induction phase and development of root primordia, BS leaves treated with IBA were harvested 12, 24 and 72 h and 10 days after the treatments. Roots were also harvested 30 days after root induction. Leaves treated and not treated with IBA were also harvested 24 h after treatments. In addition, BS leaves simultaneously treated with IBA and NPA were harvested 24 h and 10 days after the start of the experiment. In leaves treated with IBA for 5 days and then transferred to the NPA containing medium, the material was harvested 10 days after starting the treatment. As most of the roots developed from this area, the basal part of each leaf containing the petiole was embedded and frozen in Jung tissue freezing medium (Leica Microsystems, Wetzlar, Germany) on dry ice.

Cryosections (10 μm) were cut from the samples and placed on 3-aminopropyl-triethoxysilane glass slides. Cryostat sections were dried at 40 °C and fixed in 3:1 (*v*/*v*) ethanol:glacial acetic acid for 10 min followed by 5 min in 70% ethanol. A 650 bp fragment corresponding to the 5′ region of the *CsSCL1* gene (outside of the GRAS domain) was cloned into the PCR^®^ II vector (Invitrogen, Waltham, MA, USA) and amplified to generate *CsSCL1* specific probes. The PCR fragment, flanked by SP6 and T7 promoters, was used as a template for synthesizing both sense and antisense digoxigenin (DIG)-labelled probes, with, respectively, T7 and SP6 polymerase, according to the manufacturer’s instructions (Roche Biochemicals, Hoffmann, Germany). The probes were partially hydrolyzed by alkali treatment, to a mean length of 200 nucleotides. In situ hybridization was performed as described by Sánchez et al. [18], and the hybridization signal was detected using a DIG Nucleic Acid Detection kit (Roche Biochemicals, Hoffmann, Germany) according to the manufacturer’s instructions.

For histological purposes, serial cryosections (10 µm) used in in situ experiments were stained in 0.05% toluidine blue O. Photographs were taken under bright-field illumination in a Nikon microscope equipped with an Olympus digital camera. Scale bars are shown at the bottom right corner of each image to indicate magnification degree.

### 4.6. Statistical Analysis

All rooting experiments were repeated three times with 18 explants per treatment. Percentage data were subjected to arcsine transformation before statistical analysis. Rooting parameters and the qPCR data were expressed as means ± SE (standard error) from three replicates. Multiple-group comparisons were evaluated by ANOVA followed by Duncan’s test. Differences were considered to be statistically significant at *p* ≤ 0.05.

## 5. Conclusions

In conclusion, the proposed in vitro leaf system in chestnut reproduces the physiological rooting response of the mother microshoots, which in turn is correlated with the ontogenetic stage of the tree material from which the microshoots were initiated. This system provides several advantages over the experimental microshoot system for studying the adventitious root organogenesis at the molecular level. Using the proposed new system, we demonstrated the inhibitory effect of NPA on root induction and root development. In addition, the negative effect of NPA on AR was directly correlated with the reduction in the auxin-induced expression of *CsSCL1* observed in rooting competent cells. The system will also be useful for studying the effects of the chronological age of leaves on rooting ability and could also eventually be extended to other woody species.

## Figures and Tables

**Table 1:** Rooting response of P2 BS chestnut microshoots derived from basal sprouts, leaves excised from BS microshoots and leaf segments. Microshoots were dipped in 4.9 mM IBA solution for 1 min and then placed on IBA-free medium. Leaves and leaf segments were placed for 5 d on medium containing 25 µM IBA and then transferred to IBA-free medium. Each value represents the mean (±SE) from three experiments with 18 replicates of each type of explant. NBR: Non-basal roots; LRL: longest root length; MRT: Mean rooting time.

Explant	Rooting (%)	N° Roots	NBR (%)	LRL (cm)	MRT (days)
Shoot	94.8 ± 3.2 (a)	8.4 ± 0.9 (a)	51.7 ± 19.8 (a)	2.6 ± 1.8 (b)	11.3 ± 0.7 (b)
Leaf	96.3 ± 1.9 (a)	5.8 ± 0.4 (b)	8.7 ± 0.8 (b)	3.7 ± 3.1 (a)	9.3 ± 0.3 (a)
Leaf segment	69.1 ± 5.0 (b)	2.9 ± 0.2 (c)	2.9 ± 1.5 (b)	2.5 ± 0.8 (b)	11.1 ± 0.2 (b)

Means followed by the same letter in each column are not significantly different at *p* ≤ 0.05 according to Duncan’s test.

## Data Availability

Not applicable.

## Supplementary Materials

The following supporting information can be downloaded at: https://www.mdpi.com/article/10.3390/plants12142657/s1, Table S1: List of primers used for qPCR analysis, Figure S1: In vitro rooted leaves excised from microshoots established from chestnut basal sprouts. Leaves were placed abaxial side down on medium containing 25 µM IBA for 5 days and then transferred to IBA-free medium, Figure S2: Plant material excised from microshoots at the end of proliferation (a) and rooting phases (b). ab: axillary buds; ar: apical section of the root; br: basal section of the root; i: internodes; l: leaf; mr: middle section of the root, Figure S3: Influence of the ontogenetic stage on the rooting response of microshoots derived from basal shoots (BS shoot) and from crown branches (CR shoot) and of leaves excised from these microshoots.
