# Supplementary material for: Expression of CsSCL1 and Rooting Response in Chestnut Leaves Are Dependent on the Auxin Polar Transport and the Ontogenetic Origin of the Tissues
Source: Plants (Basel). 2023 Jul 16;12(14):2657. doi: 10.3390/plants12142657 (PMC10385970; doi:10.3390/plants12142657)
Supplement: Supplementary file 1 [file plants-12-02657-s001.zip › plants-2486425-supplementary.pdf]

Table S1. List of primers used in this study for qPCR analysis

Gene symbol	Accession number	Gene description	Primer sequence (5´-3´) Forward/Reverse	Amplicon length
ACT	ID6828205	*Actin 2*	GCCCCACGAGCTGTGTTC/ TCTGGCCCATTCCAACCA	73 nt
SCL1	DQ683579	*Scarecrow-like 1*	CGCCTCCTATTCTGGGTGAGTA/ GCCAACCCATCACCAAAATG	118 nt
TUB	ID2842854	*β-Tubulin*	CTCGTGCTGTTCTCATGGATCT/ TGGCCGAAAACGAAGTTGTC	100 nt
UBI	ID3924917	*Polyubiquitin 3*	AGGAATCAACCCTTCACCTTGTC/ GAACTCTCCACCTCCAAAGTGATG	100 nt

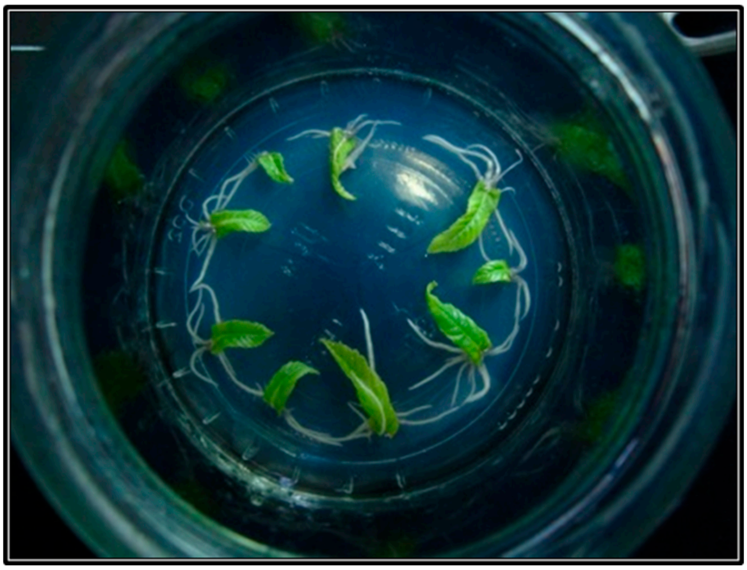

Figure S1. In vitro rooted leaves excised from microshoots established from chestnut basal sprouts. Leaves were placed abaxial side down on medium containing 25 μM IBA for 5 days and then transferred to IBA-free medium.

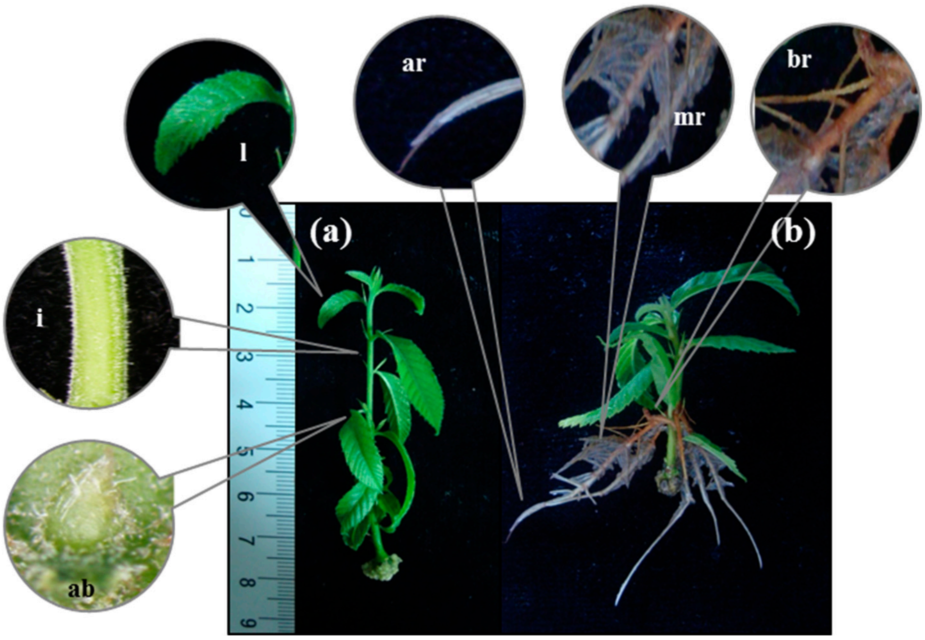

Figure S2. Plant material excised from microshoots at the end of proliferation (a) and rooting phases (b). ab: axillary buds; ar: apical section of the root; br: basal section of the root; i: internodes; l: leaf; mr: middle section of the root.

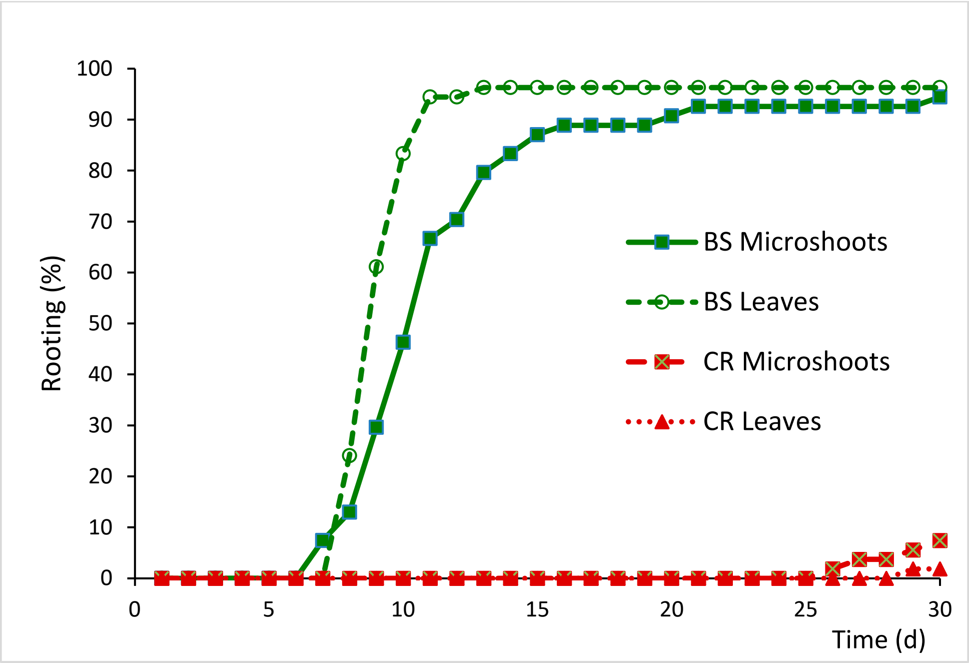

Figure S3. Influence of the ontogenetic stage on the rooting response of microshoots derived from basal shoots (BS microshoots) and from crown branches (CR Microshoots) and of leaves excised from these microshoots (BS leaves, CR Leaves).
